# Eradication of *Pseudomonas aeruginosa* biofilms and persister cells using an electrochemical scaffold and enhanced antibiotic susceptibility

**DOI:** 10.1038/s41522-016-0003-0

**Published:** 2016-11-23

**Authors:** Sujala T Sultana, Douglas R Call, Haluk Beyenal

**Affiliations:** 1 The Gene and Linda Voiland School of Chemical Engineering and Bioengineering, Washington State University, Pullman, WA 99163 USA; 2 Paul G Allen School for Global Animal Health, Washington State University, Pullman, WA 99163 USA

## Abstract

Biofilms in chronic wounds are known to contain a persister subpopulation that exhibits enhanced multidrug tolerance and can quickly rebound after therapeutic treatment. The presence of these “persister cells” is partly responsible for the failure of antibiotic therapies and incomplete elimination of biofilms. Electrochemical methods combined with antibiotics have been suggested as an effective alternative for biofilm and persister cell elimination, yet the mechanism of action for improved antibiotic efficacy remains unclear. In this work, an electrochemical scaffold (e-scaffold) that electrochemically generates a constant concentration of H_2_O_2_ was investigated as a means of enhancing tobramycin susceptibility in pre-grown *Pseudomonas aeruginosa* PAO1 biofilms and attacking persister cells. Results showed that the e-scaffold enhanced tobramycin susceptibility in *P. aeruginosa* PAO1 biofilms, which reached a maximum susceptibility at 40 µg/ml tobramycin, with complete elimination (7.8-log reduction vs control biofilm cells, *P* ≤ 0.001). Moreover, the e-scaffold eradicated persister cells in biofilms, leaving no viable cells (5-log reduction vs control persister cells, *P* ≤ 0.001). It was observed that the e-scaffold induced the intracellular formation of hydroxyl free radicals and improved membrane permeability in e-scaffold treated biofilm cells, which possibly enhanced antibiotic susceptibility and eradicated persister cells. These results demonstrate a promising advantage of the e-scaffold in the treatment of persistent biofilm infections.

## Antibiotics: Enhanced effect with an electrical scaffold

Using an electrically conductive fabric to generate hydrogen peroxide could eradicate persistent biofilms in chronically infected wounds. Electrochemical scaffolds (e-scaffolds) are thin networks of conductive material such as carbon fiber used to generate chemical responses in media they are in contact with. Haluk Beyenal and colleagues at Washington State University, USA, investigated the effect of a carbon fabric e-scaffold on cultured biofilms of the bacterium *Pseudomonas aeruginosa*. The procedure enhanced the susceptibility of this troublesome multidrug-resistant bacterium to the antibiotic tobramycin. Crucially, it eradicated so-called persister cells that can evade antibiotic treatment to reform biofilms in chronic wounds. The research suggests that the effect involves the production of hydroxyl free radicals from hydrogen peroxide and increased permeability of the bacterial cell membranes. The potential of e-scaffolds for treating infected wounds warrants further exploration.

## Introduction

Chronic and recalcitrant biofilm infections on wounds are often caused by the presence of a bacterial subpopulation of “persister cells” that are particularly tolerant to bacteriostatic antibiotics.^1^ Biofilms protect bacterial communities in part because the extracellular polymeric substances (EPS) that form the biofilm matrix serve as a diffusion barrier.^2^ This barrier limits antibiotic penetration into biofilms^3^ and immobilizes antibiotics.^4^ In addition, the diffusive barrier results in nutrient gradients, causing decreased growth and metabolic inactivity in parts of the biofilm community, which allows persister cells to arise.^1^ In particular, increased persister cell formation is observed in Gram-negative bacterial biofilms because the bacterial cell membranes are composed of lipopolysaccharides that further limit antibiotic penetration into the cells^5^.

In lieu of classical antibiotics, a number of alternative antimicrobial treatments are being explored either individually (e.g., silver,^6^ mannitol^7^) or in combination with conventional antibiotics.^8–10^ Unfortunately, high concentrations of these antimicrobials/antibiotics have toxic side effects^11,12^ while at low concentrations they often decompose before completely eliminating biofilm communities.^13^ Moreover, any persister cells can regrow and form biofilms with potentially enhanced tolerance to antibiotics.^14^ Interestingly, the application of an antibiotic in combination with a direct current (DC) (ranging from µA to mA/cm^2^) can be effective against several Gram-negative bacteria,^15,16^ including against putative persister cells.^16^ The mechanism underlying this effect was unclear until a recent study demonstrated the presence of electrochemically generated H_2_O_2_.^17^ In that study, an electrochemical scaffold (e-scaffold) made of conductive carbon fabric generated ~25 µM H_2_O_2_ near the surface of the e-scaffold and this was sufficient to reduce an *A. baumannii* biofilm (~3-log) that was established on a dermal explant. The constant but relatively low production of H_2_O_2_ did not appear to be cytotoxic to the mammalian tissue.^17^

A similar study demonstrated that electrochemically generated H_2_O_2_ is sufficient to prevent or delay *Pseudomonas aeruginosa* PAO1 biofilm growth *in vitro* ^18^ despite the production of two important catalase enzymes (*katA* and *katB*) that can protect *P. aeruginosa* from H_2_O_2_.^19^ Nevertheless, complete elimination of mature *P. aeruginosa* PAO1 biofilms by e-scaffold treatment can be difficult. Adjunct antibiotic treatment can be helpful,^16^ but the mechanism underlying this combined effect is not understood.^15^ For instance, Nodzo et al*.* reported an enhanced efficacy of vancomycin when it was combined with a cathodic potential of −1.8 V_Ag/AgCl_ against biofilms formed on Ti implants in a rodent model, but did not report any mechanism.^20^ Niepa et al*.* reported that a stainless steel (SS304) electrode released metal cations that enhanced antibiotic efficacy against *P. aeruginosa* PAO1 persister cells in an electrochemical system applying ~70 µA/cm^2^ DC.^16^ Under this condition, it is likely that SS304 corroded and released iron ions.^21^ A similar increased efficacy of antibiotic was reported when an inert carbon electrode under the same applied DC was used against *P. aeruginosa* PAO1 persister cells in the same system.^22^ An inert carbon electrode does not release metal cations as SS304 does; thus, the release of metal cations is unlikely to be the mechanism for the efficacy of a combination of DC and antibiotic treatment.^22^ The authors speculated that electrochemically generated reactive oxygen species (ROS) (e.g., H_2_O_2_ and OH•) were responsible for this effect, but they did not confirm this experimentally.^22^

An e-scaffold generates H_2_O_2_, which enters the bacterial periplasm through porins,^23,24^ where it can induce intracellular production of highly reactive hydroxyl free radicals (OH•)^25,26^ that degrade membrane lipids, proteins, and DNA.^26,27^ Recent research also found that H_2_O_2_ eliminates some of the persister cells in biofilms, facilitates the disruption of biofilm architecture and mediates the generation of metabolically active dispersal cells in a range of Gram-negative bacterial biofilms.^28,29^ Such metabolic activity in surviving dispersal cells and OH• production have been reported to induce bacterial sensitivity to antibiotic treatment.^30–32^ Therefore, e-scaffold generated H_2_O_2_ possibly promotes intracellular OH• production that in turn improves antibiotic sensitivity in biofilms and attacks persister cells.

In this work, we used *P. aeruginosa* PAO1 with an aminoglycoside antibiotic (tobramycin). *P. aeruginosa* PAO1 can resist tobramycin by producing periplasmic glucans, mutations of ribosome-binding sites or increased efflux pump action inhibiting cellular uptake.^33^ Furthermore, *P. aeruginosa* PAO1 biofilm persister cells are reportedly less sensitive to tobramycin.^9^ We isolated persister cells from *P. aeruginosa* PAO1 biofilms after treating them with ciprofloxacin following published protocols.^16,34^ We hypothesized that the bacterial subpopulation that survived e-scaffold generated H_2_O_2_ would be more sensitive to tobramycin than these ciprofloxacin-tolerant persister cells. The objectives of this study were (1) to evaluate the tobramycin susceptibility of *P. aeruginosa* PAO1 biofilms treated with an, e-scaffold and compare it with the tobramycin susceptibility of persister cells, (2) to evaluate the efficacy of the e-scaffold against persister cells and (3) to determine whether e-scaffold treatment would increase intracellular production of OH• radicals and increase membrane permeability in the bacterial cells, making them more susceptible to antibiotics. In addition, change in bacterial cell morphology after e-scaffold treatment was observed using scanning electron microscopy (SEM). Finally, based on these observations, a possible mechanism of e-scaffold enhanced antibiotic susceptibility is proposed and a future mechanistic study is suggested.

## Results

### Electrochemical scaffold enhances tobramycin susceptibility in biofilm cells

**Fig. 1:**
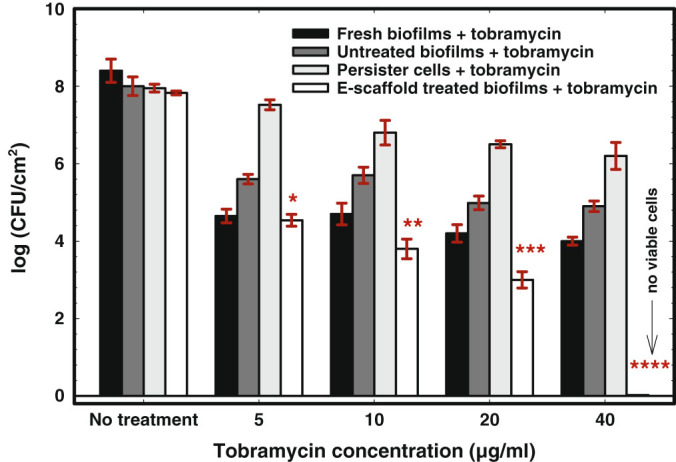
E-scaffold enhances tobramycin susceptibility in *P. aeruginosa* PAO1 biofilms. Bars represent means of logarithms of colony-forming units of viable biofilm cells. Error bars represent the standard error of the means from three biological replicates. The symbols *, **, ***, and **** represent significant differences in tobramycin susceptibility between e-scaffold treated biofilms + tobramycin and untreated biofilms + tobramycin (*n* = 3, *, *P* = *0.002*; **, ***, **** *P* ≤ 0.001; paired *t*-test)

When biofilm treatments were combined with different concentrations of tobramycin, the surviving cells responded differently. The tobramycin susceptibility of *P. aeruginosa* PAO1 biofilms regrown from fresh culture, untreated biofilm cells, and persister cells isolated from biofilms appeared to follow a dose response at tested concentrations between 0 and 40 µg/ml (Fig. 1). The biofilms regrown from persister cells showed tolerance to tobramycin, with only a (1.2 ± 0.16)-log reduction in viable cells for 10 µg/ml tobramycin and no further significant decrease at higher concentrations. These persister cells had the same minimum inhibitory concentration (MIC) as the fresh culture (Supplementary information); however, consistent with the characteristic behavior of “persister cells”, they survived antibiotic treatment, regrew and developed tolerance to tobramycin identical to their regular population.^1,35^ Interestingly, tobramycin tolerance was observed in biofilms regrown from untreated biofilm cells and persister cells isolated from biofilms. In contrast, no tolerance to tobramycin was identified for biofilms regrown from e-scaffold treated cells when different concentrations of tobramycin were combined with e-scaffold treatment (Fig. 1). This confirms the prevention of persistence by the e-scaffold. A linear dose response was observed for the log reduction of e-scaffold treated biofilm cells which received tobramycin treatment, leading to a complete eradication at 40 µg/ml tobramycin (Fig. 1). This concentration (20×MIC) is still considerably lower than what is typically required for *P. aeruginosa* PAO1 biofilm treatment with tobramycin (>500× of MIC) as reported in the literature.^36^ Overall, a significant increase in tobramycin susceptibility was attained for e-scaffold treated biofilms compared to biofilms that were not treated with an e-scaffold (*P* < 0.05, paired t-tests) (Fig. 1). Among the tested tobramycin concentrations, we observed a maximum tobramycin susceptibility at 40 µg/ml in e-scaffold treated biofilms compared to that for persister cells.

### Electrochemical scaffold eradicates persister cells

**Fig. 2:**
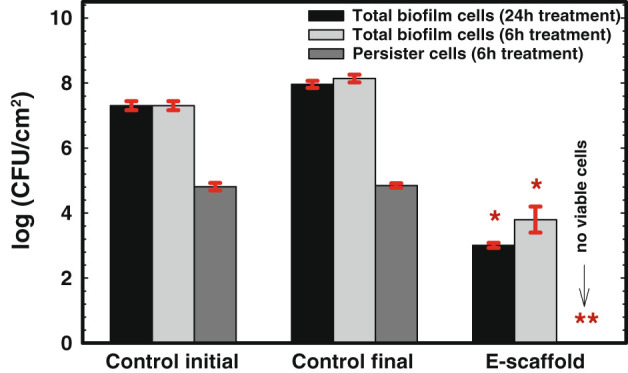
Exposure to e-scaffold eradicates the persister cells in *P. aeruginosa* PAO1 biofilms. Bars represent means of logarithms of colony-forming units of viable biofilm cells. Error bars represent the standard errors of the means from three biological replicates. The symbol * denotes a significant difference compared to total biofilm cells (*n* = 3, *P* < 0.05, one-way ANOVA), and ** denotes a significant difference compared to control initial persister cells (*n* = 3, *P* < 0.001*,* one-way ANOVA)

The effect of e-scaffold generated H_2_O_2_ on *P. aeruginosa* PAO1 persister cells isolated from ciprofloxacin-treated biofilms was explored. As Fig. 2 shows, ~0.31 % of the total biofilm cells belonged to the persister population, which is in the range reported in the literature.^37^ Within 6 h of these persister cells being treated with an e-scaffold, no viable persister cells were apparent. This corresponds to a 5-log reduction in persistence compared to the control initial persister cells (*P* ≤ 0.001*,* one-way ANOVA followed by Bonferroni test). No growth was observed even after the treated cells were exposed to fresh medium alone for 24 h upon removal of the e-scaffold. This confirmed complete eradication of persister cells by e-scaffold treatment. In contrast, the final population of persister cells did not change within the additional 6 h of ciprofloxacin treatment. The e-scaffold was also found to be effective against total biofilm cells, with (4.2 ± 0.23) and (4.95 ± 0.20)-log reductions in viable cells within 6 and 24 h of treatment, respectively, compared to the control final biofilm cells. Together these results suggest that the e-scaffold is effective against both regular biofilm cells in active states and persister cells in inactive metabolic states. They also confirm that persister cells are inherently more sensitive to e-scaffold generated H_2_O_2_ exposure.

### E-scaffold induces OH• generation and increases membrane permeability

**Fig. 3:**
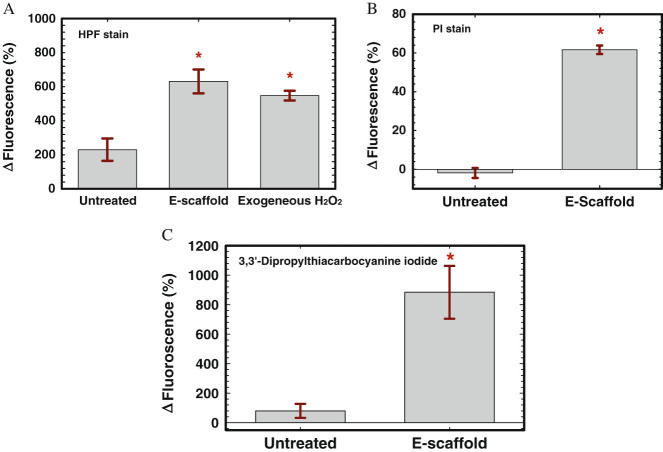
E-scaffold increases OH• formation and membrane permeability. **a** Increase in fluorescence of HPF-stained e-scaffold treated and exogenous H_2_O_2_ treated *P. aeruginosa* PAO1 biofilm cells indicates increased OH• formation compared to untreated biofilms. **b** Increase in fluorescence of propidium iodide (PI) indicates increased membrane permeability of *P. aeruginosa* PAO1 cells after exposure to e-scaffold. **c** Increase in fluorescence of 3, 3′-dipropylthiacarbocyanine iodide, a membrane potential sensitive dye, in e-scaffold treated *P. aeruginosa* PAO1 cells verifies bacterial membrane depolarization by e-scaffold resulting in increased membrane permeability. Error bars represent standard errors of means for at least three biological replicates. The symbol * indicates a significant difference from the untreated biofilm cells (*n* = 3, *P* < 0.001; paired *t-*test)

We observed increased fluorescence for both e-scaffold treated and exogenous H_2_O_2_ treated biofilm cells, indicating enhanced OH• formation after e-scaffold generated H_2_O_2_ treatment (Fig. 3a). Furthermore, an increase in the propidium iodide (PI) fluorescence of e-scaffold treated cells was observed compared to untreated biofilm cells (Fig. 3b). PI is a membrane-impermeable dye and can only enter a bacterial cell if the outer membrane is damaged. It can permeate the cytoplasm and bind to the DNA, showing an increased fluorescence intensity, only upon disruption of the cell membranes by the e-scaffold.^22,38^ Thus, an increase in PI intensity corresponds to an increased membrane permeability. Figure 3b shows that PI permeability increased in e-scaffold treated cells, indicating possible damage to the outer cell membrane by the e-scaffold. Increased membrane permeability was additionally verified using 3,3′-dipropylthiacarbocyanine iodide, a membrane potential sensitive dye. An increase in the fluorescence intensity of 3,3′-dipropylthiacarbocyanine iodide was observed in e-scaffold treated cells compared to untreated biofilm cells (Fig. 3c), indicating membrane depolarization after exposure to e-scaffold. The dissipation of the membrane potential may be caused by the perturbation of the membrane lipid bilayers.^39^ Membrane potential sensitive dye 3,3′-dipropylthiacarbocyanine iodide enters only depolarized cells, where it binds reversibly to lipid-rich intracellular components.^40^ The increased fluorescence intensity of this dye in e-scaffold treated cells (Fig. 3c) indicates that the e-scaffold disrupted the cytoplasmic membrane and that this induced depolarization of the plasma membrane potential. Thus Fig. 3 suggests that the e-scaffold increased membrane permeability, possibly upon the membrane integrity of the cells being compromised by OH•.

**Fig. 4:**
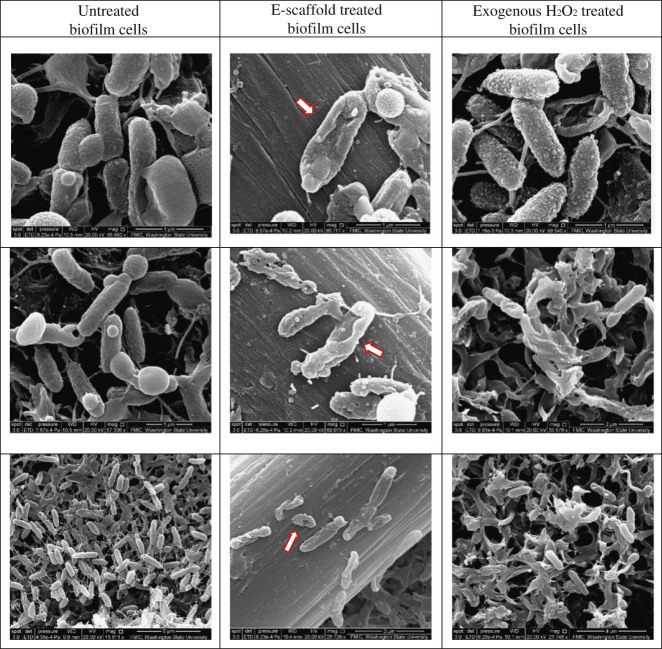
Scanning electron microscopy (SEM) images showing untreated, e-scaffold treated and exogenous H_2_O_2_ treated *P. aeruginosa* PAO1 biofilm cells. Three representative images are shown for each treatment. Red arrows indicate cells showing a stressed membrane

Additionally, membrane-compromised cells similar to those in exogenous H_2_O_2_ treated samples were observed in e-scaffold treated samples in SEM images (Fig. 4). Similar deformation was observed previously by Istanbullu et al. in cells treated with electrochemically generated H_2_O_2_^18^ and by DeQueiroz et al*.* and Diao et al*.* in cells treated with exogenous H_2_O_2_.^41^ Untreated biofilm cells showed intact cell walls and membranes (Fig. 4). Thus, Fig. 4 indicates e-scaffold generated H_2_O_2_ induced morphological changes in cells and structural damage of the outer membrane that might increase membrane permeability.^42,43^

## Discussion

Electrochemical method combined with antibiotic has been suggested to be effective against biofilms in several previous studies, yet the mechanism behind this increased antibiotic efficacy remains unclear.^15,44^ In this work, an e-scaffold that electrochemically generates a constant concentration H_2_O_2_ was investigated as a mean of enhancing antibiotic efficacy against biofilm cells and its efficacy against ciprofloxacin-tolerant persister cells was evaluated. Overall, our results indicate that the e-scaffold induced intracellular formation of OH• and improved membrane permeability. These mechanisms enhanced tobramycin efficacy, including against persister cells. 

**Fig. 5:**
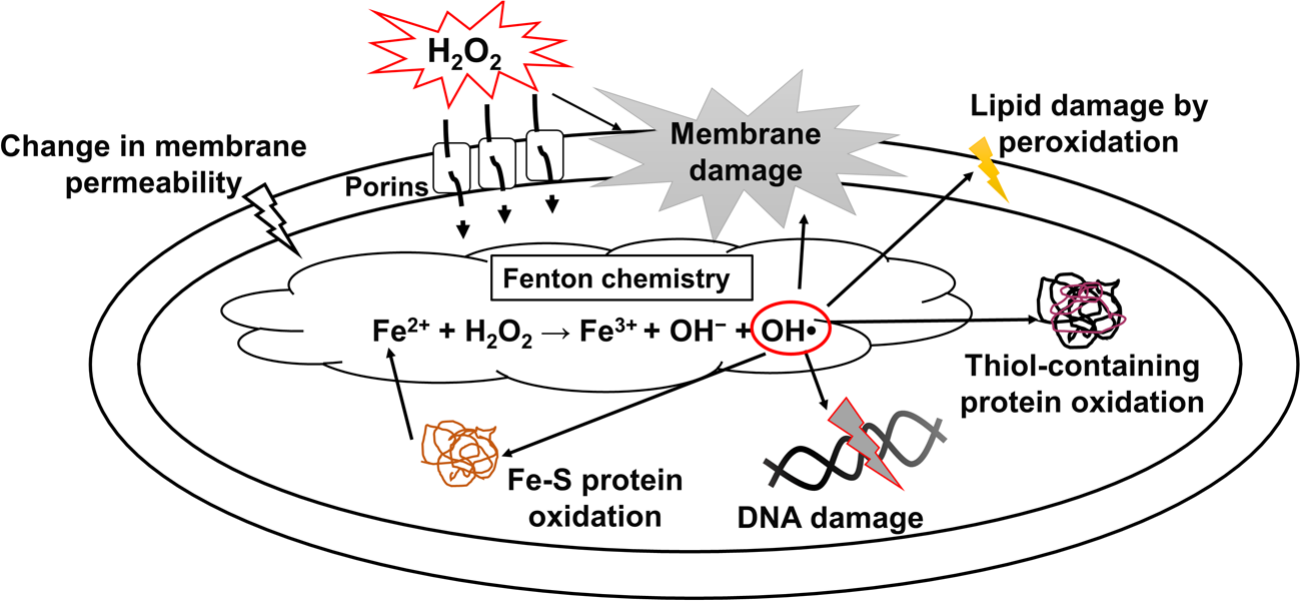
Proposed mechanism for e-scaffold generated H_2_O_2_ enhancing antibiotic susceptibility. H_2_O_2_ generated extracellularly by e-scaffold diffuses through the bacterial cell envelope and reacts with intracellular Fe^2+^, forming radicals that oxidize lipids, proteins and DNA, which prompts cell death. Through membrane damage or changing membrane permeability, antibiotic penetration into the bacterial cell is increased, which in turn enhances the antibiotic susceptibility of the cells

The biofilm removal mechanism of e-scaffolds is electrochemical generation of H_2_O_2_, which is a potential biocide and oxidizing agent.^17^ H_2_O_2_ mediates dispersal in biofilms, disrupts various bacterial processes and cellular networks, and disrupts the cell envelope through intracellular production of ROS such as OH• in Gram-negative bacteria, as shown in Fig. 5.^26,28,45^ Our observations suggest that e-scaffold generated H_2_O_2_ increases intracellular OH• formation in Gram-negative *P. aeruginosa* PAO1 biofilm cells. Furthermore, in membrane permeability assays and SEM image analysis, we observed increased permeability with moderate membrane damage in cells after e-scaffold treatment. Thus, we propose that when e-scaffold generated H_2_O_2_ enters a bacterial cell, it induces intracellular ROS production such as OH•, which can increase the permeability of bacterial membranes.^26,27^ Increased permeability can facilitate better antibiotic penetration. These effects can potentiate the tobramycin susceptibility of *P. aeruginosa* PAO1 biofilms and eradicate persister cells in various metabolic states.^31,46^

That the e-scaffold increases the permeability of the outer membrane of Gram-negative bacteria opens up the possibility of enhancing susceptibility to a range of antibiotics. As noted above, recent papers questioned the role of electric current in electrochemically generating H_2_O_2_ that enhanced antibiotic susceptibility in biofilms and associated persister cells.^15,22,47^ It has already been confirmed that an e-scaffold at a constant applied potential (−600 mV_Ag/AgCl_) electrochemically generates H_2_O_2_, which is the mechanism of action for biofilm removal.^17^ Here, we explained that e-scaffold generated H_2_O_2_ can lead to intracellular OH• production in bacteria that possibly enhances the efficacy of tobramycin against *P. aeruginosa* PAO1 biofilms and thus eradicates biofilms and associated persister cells.

The observed effect of increased intracellular OH• formation as a possible mechanism for enhanced antibiotic susceptibility in bacteria needs to be further confirmed by more mechanistic studies. For instance, decreasing OH• through the addition of thiourea (150 mM), an OH• scavenger,^48^ inhibited bacterial cell death due to e-scaffold and tobramycin (Fig. S2, Supplementary information), indicating that OH• production may be critical for the observed bactericidal activity. Additionally, overexpression of cellular ROS scavengers such as superoxide dismutase (SOD) can be used to inhibit e-scaffold induced OH• formation and bacterial cell death. Thus, it can be verified whether OH• formation is a critical factor for the observed bacterial killing by e-scaffold and tobramycin. As indicated in Fig. 5, OH• formation occurs through the Fenton reaction and Fe^2+^ plays a vital role in this reaction. Testing the efficacy of e-scaffold and tobramycin against mutant strains with impaired iron regulation would be another way to confirm OH• formation as the fundamental mechanism for e-scaffold enhanced antibiotic susceptibility.^38^

Finally, this research proposes future use of the e-scaffold as an effective individual or adjuvant therapy against persistent biofilm infections. Notably, the application of AC and DC electric fields combined with antibiotics involves a different mechanism^49^ than that of the electrochemical biofilm control method proposed in this work.

## Materials and methods

### Growth medium, chemicals, and antibiotics

For all experiments, 20 g/L (1×) Luria broth (LB) medium (Sigma-Aldrich, catalog #L3522) was used to grow overnight *P. aeruginosa* PAO1 cultures and 1 g/L (0.05×) LB was used as the growth medium for biofilms. Tobramycin (Sigma Aldrich, catalog #T4014) and ciprofloxacin (Sigma Aldrich, catalog #17850) solutions were diluted in 1 g/L (0.05×) LB for antibiotic susceptibility experiments and persister cell isolation, respectively. Minimum inhibitory concentrations (MICs) were determined for both antibiotics following the Clinical and Laboratory Standards Institute (CLSI) protocol as detailed in Supplementary information.^50^ Other key compounds included 3′-p-hydroxyphenyl fluorescein dye (Invitrogen, catalog #H36004), propidium iodide (Invitrogen, catalog #L-7012), 3, 3′-dipropylthiacarbocyanine iodide (Sigma Aldrich, catalog #318434), thiourea (AK Scientific, Inc., catalog #S726) and H_2_O_2_ (VWR, catalog #RC3819-16).

### Culture and biofilm preparation

**Fig. 6:**
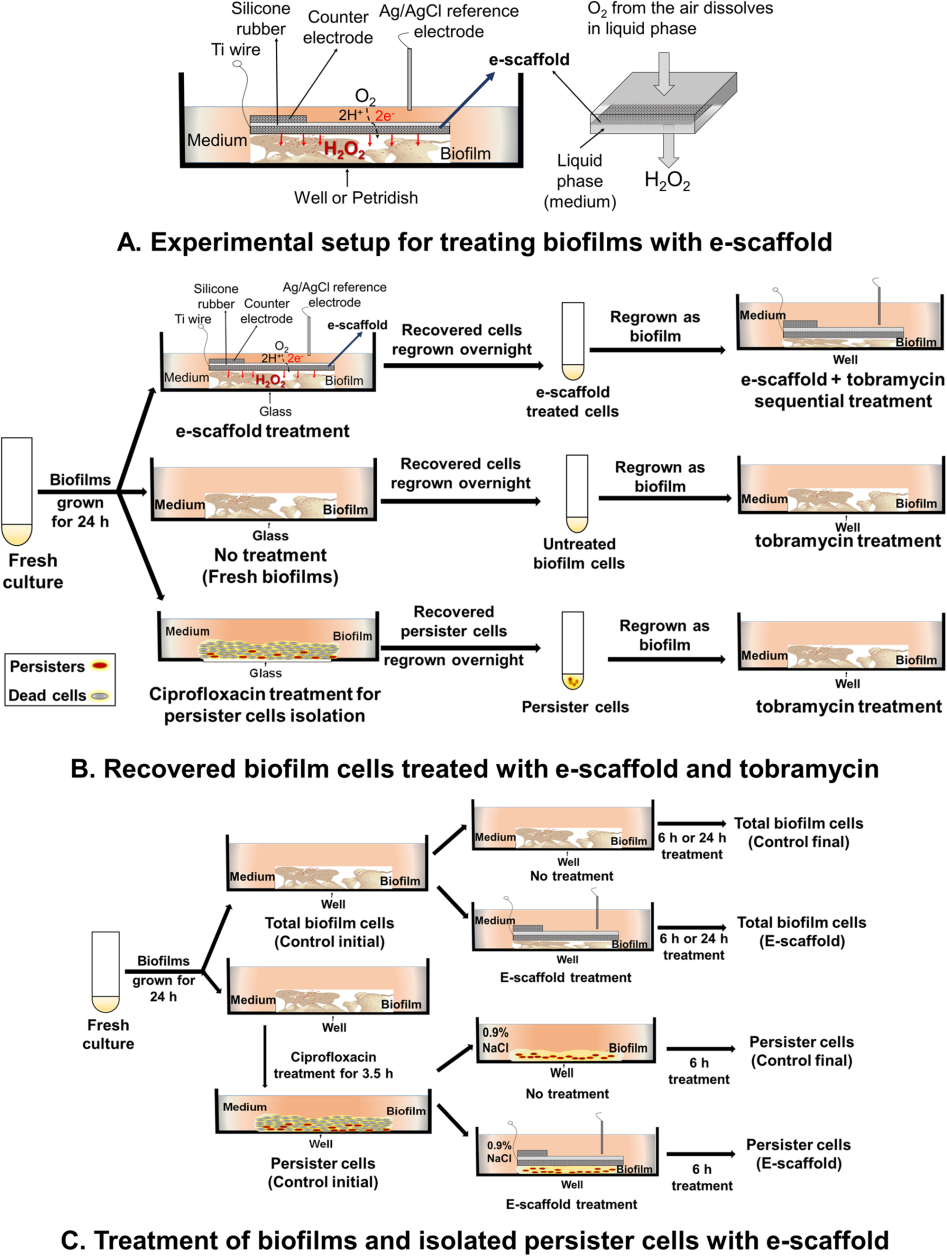
**a** Schematic of experimental setup for the treatment of biofilm exposed to an e-scaffold with an illustration of electrochemical H_2_O_2_ production. The electrodes are connected to a potentiostat (not shown in figure). Scientific Reports, 2015. 5, 14908. DOI: 10.1038/srep14908. © Creative Commons Attribution 4.0 International License. **b** Recovered biofilm cells treated with e-scaffold and tobramycin. **c** Treatment of biofilms and persister cells with e-scaffold

Frozen stocks of *P. aeruginosa* PAO1 were cultured overnight in LB at 37 °C on a rotating table (70 rpm). For biofilm experiments, overnight culture was adjusted to OD_600 _≈ 0.5 in LB and used as inoculum.^17^ Briefly, 2 ml of culture was used to inoculate sterile glass bottom petri dishes (MatTek Corporation, catalog #P35G-1.5-20-C) and allowed to form biofilms for 24 h. These one-day-old biofilms were treated with e-scaffolds for 24 h, as described previously.^17^ Briefly, untreated and e-scaffold treated biofilm cells were washed twice to remove loosely attached cells and then remaining cells were recovered for antibiotic susceptibility testing. To identify and isolate *P. aeruginosa* PAO1 persister cells, planktonic culture was grown to the stationary phase and the dose-dependent killing curve for ciprofloxacin (0–200 μg/mL) was investigated (Supplementary information, Fig. S1).^34^ A plateau of the surviving subpopulation was observed for ciprofloxacin concentrations above 50 μg/mL (200× of MIC), and these were identified as persister cells.^34,51^ We assessed the antibiotic treatment on biofilms from three different populations: cells recovered after no treatment (“untreated biofilms”), cells recovered after e-scaffold treatment (“e-scaffold treated biofilm”) and “persister cells” that were isolated from biofilms treated with 200 μg/mL ciprofloxacin for 3.5 h according to a published protocol.^34^ For experiments with persister cells, the ciprofloxacin-treated biofilms were washed and refreshed with 0.9 % NaCl for subsequent experiments. This method for culture, biofilm preparation and treatments is illustrated in Fig. 6.

### Biofilm treatment with e-scaffold

*P. aeruginosa* PAO1 biofilms were exposed to e-scaffold treatment for 24 h, as described previously.^17^ Briefly, a custom-built e-scaffold was fabricated using carbon fabric as detailed in Supplementary information.^17^ Biofilms were carefully washed (2×) with LB (0.05×) and an e-scaffold was overlaid onto the biofilms followed by 4 mL of fresh medium. A standard Ag/AgCl (saturated KCl) reference electrode was introduced to apply a constant potential (-600 mV_Ag/AgCl_) to an e-scaffold using a Gamry Series G 300 potentiostat (Gamry Instruments, Warminster, PA, USA) to reduce oxygen, generate a low concentration of H_2_O_2_ and deliver it continuously to the biofilm*s* (Fig. 6a). After treatment, the viable cell counts were determined using a modified drop-plate cell counting method.^17,52^ Loosely attached cells were removed by carefully washing the biofilms with 0.9 % NaCl before they were resuspended in 5 mL of 0.9 % NaCl and vortexed for 30 s. These suspensions were centrifuged (4180 × g for 10 min), and the resulting cell pellet was resuspended in 1 mL of 0.9 % NaCl. Aliquots (250 µL) were then serially diluted, and 10 µL of each dilution was plated onto LB agar. Plates were incubated for 24 h (37 °C), and colony-forming units (CFU) were enumerated.

### Tobramycin susceptibility of e-scaffold treated cells

To determine whether e-scaffold treatment altered susceptibility to antibiotics,^35^ biofilm treatments were combined with tobramycin treatments. For this experiment, the recovered biofilm cells were harvested (Fig. 6b) and adjusted to OD_600 _≈ 0.5 in LB medium (0.05×). A 1-mL cell suspension was used to inoculate a 24-well plate, where biofilms were allowed to form. Biofilms were treated with an e-scaffold for 2 h. Each well was then washed (2×) and challenged with 1 mL of one of the test concentrations (5, 10, 20 and 40 µg/mL) of tobramycin for 6 h. After treatment, the cells were washed (2×), resuspended in 0.9 % NaCl and processed to enumerate CFU.^52^ Cell counts were compared for e-scaffold and other treated and untreated biofilms. The treatments are summarized in Fig. 6b.

### Effect of e-scaffold on persister cells

Biofilms were grown for 24 h in 6-well plates and treated with 200 μg/mL ciprofloxacin for 3.5 h to isolate persister cells from ciprofloxacin-treated biofilms.^16^ The total number of viable cells was determined for ciprofloxacin-treated and untreated biofilms using the modified drop-plate cell counting method.^52^ Remaining persister cells were then exposed to either 200 μg/ml ciprofloxacin or e-scaffold treatment for 6 h (in the 0.9 % NaCl solution). Final CFU numbers were then determined following the procedure described above. Figure 6c summarizes the treatment methodology we used.

### Hydroxyl free radical detection assay

Intracellular hydroxyl free radical (OH•) formation was detected using 5 µM of a fluorescent reporter dye, 3′-(p-hydroxyphenyl fluorescein) (HPF) (Invitrogen, catalog #H36004) following a published protocol.^38^ Briefly, e-scaffold treated, exogenous H_2_O_2_-treated and untreated biofilm cells were vortexed in 500 µL of LB in a microcentrifuge tube for 30 s. These samples were centrifuged (10,000 × *g* for 10 min), and then the medium was replaced with a final concentration of 5 µM HPF prepared in 500 µL of 0.1 M PBS. After staining in the dark at room temperature for 15 min, samples were centrifuged at 10,000 × *g* for 10 min. Supernatant was removed, and cells were rinsed and resuspended with PBS. An aliquot (100 µL) was added to each well in a 96-well plate, and fluorescence intensity was quantified using a microplate reader (Bio-Tek Cytation 5) with excitation at 490 and emission at 515 nm. For the OH• formation assays, fluorescence was estimated as follows: ((Fluorescence with dye−Fluorescence without dye)/(Fluorescence without dye))×(100).

### Membrane permeability

Change in bacterial membrane permeability was evaluated by analyzing the influx of a membrane-impermeable dye, propidium iodide **(**PI)^22,38^ according to the manufacturer’s instructions (Invitrogen, catalog #L-7012). E-scaffold treated and untreated biofilm cells were centrifuged (6000 rpm, 10 mins) to cell pellets and supernatant was poured off. The cell pellets were stained with PI for 15 min in the dark, then washed twice with 0.9 % NaCl to remove any unbound dye. Cells were then resuspended in 0.9 % NaCl, and 100 µL of each suspension was transferred in triplicate into wells of a 96-well plate. Fluorescence intensity was quantified (excitation 535 nm, emission 517 nm). Fluorescence was determined as a percentage change compared to untreated sample using the following formula: ((Fluorescence with dye−Fluorescence without dye)/(Fluorescence without dye))×(100).

Additionally, the effect of e-scaffold treatment on bacterial membrane permeability was detected using a membrane potential sensitive dye, 3, 3′-dipropylthiacarbocyanine iodide. The fluorescence intensity of 3, 3′-dipropylthiacarbocyanine iodide changes in response to changes in plasma membrane potential upon structural damage.^42,53^ Untreated biofilm cells were washed and then resuspended in buffer A (20 mM glucose, 5 mM HEPES, pH = 7.2) with 0.1 M KCl. To achieve a stable signal, 3,3′-dipropylthiacarbocyanine iodide was added to untreated and e-scaffold treated biofilm cell suspensions and incubated for 10 min. Changes in the fluorescence intensity induced by changes in membrane potential after exposure to e-scaffold were measured at an excitation wavelength of 622 nm and an emission wavelength of 670 nm.^42^ The measurements were performed in triplicate for three biological replicates in wells of a 96-well plate. Fluorescence was determined as a percentage change compared to untreated sample using the following formula: ((Fluorescence with dye−Fluorescence without dye)/(Fluorescence without dye))×(100).

### Scanning electron microscopy

For scanning electron microscopy (SEM) imaging, biofilms were grown for 24 h on UV-sterilized, 0.22-µm type GV membrane filters (Millipore, catalog ID #551200401) placed in sterile 6-well plates. Exogenous H_2_O_2_ was added continuously at an average 0.008 mmol/h for 24 h to mimic the e-scaffold generated H_2_O_2_ treatment described previously.^17^ After treatment for 24 h, both e-scaffolds and membrane filters with biofilms were aseptically collected from the untreated, e-scaffold treated and exogenous H_2_O_2_ treated wells. The membrane filters and e-scaffolds were fixed overnight with 2.5 % glutaraldehyde and 2 % paraformaldehyde in 0.1 M phosphate buffer, followed by rinsing with 0.1 M phosphate buffer (3 × 10 min each). The membranes and e-scaffolds were then dehydrated gradually by being washed sequentially with 10, 30, 50, 70, and 95 % alcohol (10 min each) and 100 % alcohol (3 × 10 min each). Hexamethyldisilizane (HMDS) was used for overnight drying. Samples were then sputter-coated with gold prior to field emission in-lens scanning electron microscopy (FEISEM) (FEI 200F) imaging.

### Statistical analysis

All experiments were conducted for at least three biologically independent replicates. Technical replicates were averaged to produce replicate means that were subsequently used for analysis. Mean values were compared within and between groups using one-way ANOVA followed by Bonferroni’s post hoc test for individual comparisons. Differences were considered statistically significant if *P* < 0.05 (Sigma plot, version 13, Systat Software, Inc., San Jose, CA).

## Conclusions

In this work, we found that *P. aeruginosa* PAO1 biofilms treated with e-scaffolds do not develop tolerance to tobramycin; thus, e-scaffolds reduce persistence. Our results indicate that the e-scaffold enhances tobramycin susceptibility in *P. aeruginosa* PAO1 biofilms and eradicates isolated persister cells. This appears to be a promising advantage of the e-scaffold for controlling persister cells. We further showed that the e-scaffold induces intracellular OH• formation and causes an increase in membrane permeability and moderate morphological changes in the bacterial membrane. We propose these effects as the mechanism by which it enhances tobramycin efficacy. These results demonstrate the potential of the e-scaffold as an alternative to conventional antibiotic treatment or as an adjuvant therapy to enhance antibiotic susceptibility in complex and persistent biofilm infections.

## Electronic supplementary material

Supplementary Information
