# Supplementary material for: Eradication of Pseudomonas aeruginosa biofilms and persister cells using an electrochemical scaffold and enhanced antibiotic susceptibility
Source: NPJ Biofilms Microbiomes. 2016 Nov 23;2:2. doi: 10.1038/s41522-016-0003-0 (PMC5460242; doi:10.1038/s41522-016-0003-0)
Supplement: Supplementary file 1 — Supplementary Information [file 41522_2016_3_MOESM1_ESM.docx]

Supplementary information for

**Eradication of *Pseudomonas aeruginosa* biofilms and persister cells using an electrochemical scaffold and enhanced antibiotic susceptibility**

Sujala T Sultana^1^, Douglas R Call^2^, and Haluk Beyenal^1,*^

^1^The Gene and Linda Voiland School of Chemical Engineering and Bioengineering, Washington State University, Pullman, WA 99163, USA

^2^Paul G. Allen School for Global Animal Health, Washington State University, Pullman, WA 99163, USA

^*^Corresponding author:

Email: beyenal@wsu.edu; Telephone: +1-509-335-6607; Fax: +1-509-335-4806

**Electrochemical scaffold preparation.** In this work, the purpose of the electrochemical scaffold (e-scaffold) working electrode was to hold a negative polarity to reduce atmospheric oxygen and generate H_2_O_2_ ^1^. To complete the electrochemical cell, we used a counter electrode and a custom-made Ag/AgCl reference electrode. A custom-built e-scaffold was fabricated using carbon fabric (Panex 30 PW-06, Zoltex Companies Inc., St Louis, MO). The fabric was cut into a circular shape (6.42 cm^2^) to serve as the e-scaffold, and a smaller circular carbon fabric “patch” (2.14 cm^2^) was used as the counter electrode. The counter electrode was attached to the e‑scaffold using a thin layer (~1 mm) of silicone rubber (DAP Dynaflex 230 Premium Indoor/Outdoor Sealant, catalog #18357), which provided insulation between the electrodes while still allowing oxygen to diffuse to the bottom surface of the e‑scaffold for H_2_O_2_ generation. For the controlled generation of H_2_O_2_, precise, accurate control of the potential of the e‑scaffold is essential ^2^, and this was achieved using a Gamry Series G 300™ potentiostat (Gamry Instruments, Warminster, PA, USA) in conjunction with a saturated Ag/AgCl reference electrode ^3^. Ti wires (0.025 Ti, Malin Co., Cleveland, OH, Lot #27567) were used as external connections to the potentiostat, and the connection resistance was consistently <2 Ω. The e‑scaffold was overlaid onto biofilms grown *in vitro*. This configuration allowed the ventral surface of the e‑scaffold to be exposed directly to biofilms.


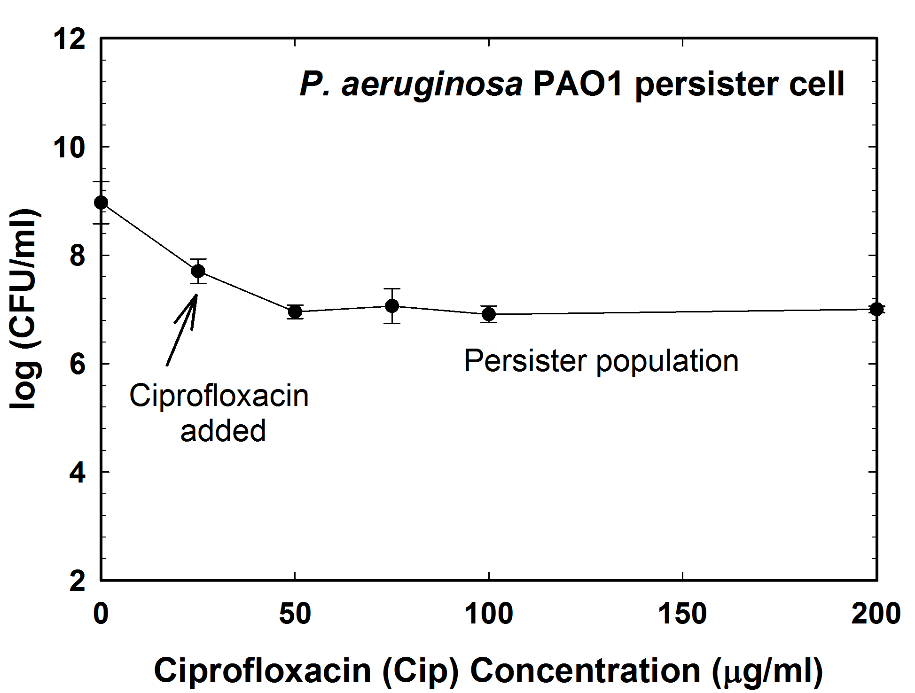


Fig. S. 1. Viability of *P. aeruginosa* PAO1 cells after a 3.5‑h treatment of a planktonic stationary phase culture. The majority of *P. aeruginosa* PAO1 cells were killed by less than 50 μg/mL of ciprofloxacin, while a small portion survived the challenge of ciprofloxacin at concentrations up to 200 μg/mL. Thus. 200 μg/mL ciprofloxacin was chosen to eliminate regular cells completely from biofilms grown for 24 h and isolate biofilm-associated control persister cells for further experiments ^4,5^. Error bars represent standard errors of means for at least three biological replicates.

**Determining minimum inhibitory concentration.** Overnight cultures were prepared from untreated biofilm cells, persister cells from ciprofloxacin-treated biofilms and e-scaffold treated cells. Cultures were grown to the stationary phase (OD_600_ ≈ 1) ^6^, then diluted to OD_600_ ≈ 0.01, and the minimum inhibitory concentrations (MICs) for ciprofloxacin and tobramycin were estimated ^7^. Briefly, 50‑μl aliquots of diluted culture were challenged with serial 2-fold dilutions of ciprofloxacin and tobramycin in 96-well plates and cultured 20 hrs. Culture without antibiotic was used as a growth control, and cell-free medium was considered a sterile control. The MIC was considered the lowest antibiotic concentration sufficient to inhibit growth compared to the fresh culture.

The tobramycin and ciprofloxacin MICs for fresh culture, e-scaffold treated biofilm cells and persister cells isolated from ciprofloxacin-treated biofilms were essentially identical (≈ 2 µg/ml for tobramycin and 0.25 µg/ml for ciprofloxacin), which confirms that biofilm cells from these treatments were not inherently more resistant to these antibiotics.

Table S. 1. Treatment and terminology for tobramycin susceptibility in biofilms

| **Culture source for biofilms**  (as illustrated in Figure 1) | **Terminology** | **Treatment** |
| --- | --- | --- |
| Fresh culture | Fresh biofilms + Tobramycin | No treatment |
|  |  | Tobramycin only (5–40 µg/ml) |
| Untreated biofilm cells | Untreated biofilms + Tobramycin | No treatment |
|  |  | Tobramycin only (5–40 µg/ml) |
| Persister cells | Persister cells + Tobramycin | No treatment |
|  |  | Tobramycin only (5–40 µg/ml) |
| E-scaffold treated cells | E-scaffold treated biofilms + Tobramycin | E-scaffold treatment for 2 h |
|  |  | Tobramycin (5–40 µg/ml) |

Table S. 2. Treatment and terminology for e-scaffold against persister cells in biofilms

| **Terminology** | **Treated cells in biofilms** | **Treatment** |
| --- | --- | --- |
| Control initial | Total biofilm cells (grown 24 h) | No treatment |
|  | Persister cells (isolated from total biofilm cells) | 200 µg/ml ciprofloxacin |
| Control final | Total biofilm cells (grown 24 h) | No treatment (after 24 h) |
|  | Persister cells (isolated from total biofilm cells) | 200 µg/ml ciprofloxacin (after 6 h) |
| E-scaffold | Total biofilm cells (grown 24 h) | E-scaffold (after 24 h) |
|  | Persister cells (isolated from total biofilm cells) | E-scaffold (after 6 h) |

Fig. S. 2. Scavenging OH• within the cell during tobramycin (Tob) treatment with e-scaffold in combination with150 mM thiourea inhibited cell death in *P. aeruginosa* PAO1 biofilms. Error bars represent standard errors of means for at least three biological replicates. No statistically significant change in viable cells was observed in biofilms treated with tobramycin with or without e-scaffold in the presence of thiourea as an OH• scavenger.

**References**

1 Sultana, S. T. *et al.* Electrochemical scaffold generates localized, low concentration of hydrogen peroxide that inhibits bacterial pathogens and biofilms. *Scientific Reports* **5**, 14908, doi:10.1038/srep14908

<http://www.nature.com/articles/srep14908#supplementary-information> (2015).

2 Istanbullu, O., Babauta, J., Hung Duc, N. & Beyenal, H. Electrochemical biofilm control: mechanism of action. *Biofouling* **28**, 769-778, doi:10.1080/08927014.2012.707651 (2012).

3 Lewandowski, Z. & Beyenal, H. *Fundamentals of Biofilm Research*. (CRC Press, 2013).

4 Niepa, T. H. R., Gilbert, J. L. & Ren, D. Controlling Pseudomonas aeruginosa persister cells by weak electrochemical currents and synergistic effects with tobramycin. *Biomaterials* **33**, 7356-7365, doi:10.1016/j.biomaterials.2012.06.092 (2012).

5 Cañas-Duarte, S. J., Restrepo, S. & Pedraza, J. M. Novel Protocol for Persister Cells Isolation. *PLoS ONE* **9**, e88660, doi:10.1371/journal.pone.0088660 (2014).

6 Mulcahy, L. R., Burns, J. L., Lory, S. & Lewis, K. Emergence of Pseudomonas aeruginosa strains producing high levels of persister cells in patients with cystic fibrosis. *J Bacteriol* **192**, 6191-6199, doi:10.1128/jb.01651-09 (2010).

7 Cockerill, F. *Methods for dilution antimicrobial susceptibility tests for bacteria that grow aerobically: approved standard*. (Clinical and Laboratory Standards Institute, 2012).
